# Cardiac safety analysis of first-line chemotherapy drug pegylated liposomal doxorubicin in ovarian cancer

**DOI:** 10.1186/s13048-022-01029-6

**Published:** 2022-08-16

**Authors:** Xin-Ru Li, Xing-Han Cheng, Guo-Nan Zhang, Xiao-Xin Wang, Jian-Ming Huang

**Affiliations:** 1 Department of Oncology, The First Affiliated Hospital of Dalian Medical University, Dalian, Liaoning, 116011 People’s Republic of China; 2 Department of Gynecological Oncology, the Affiliated Cancer Hospital, School of Medicine, University of Electronic Science and Technology of China, Sichuan Cancer Hospital & Institute, No.55 Ren-min-nan Road, Chengdu, 610000 Sichuan Province People’s Republic of China; 3 Department of Biochemistry & Molecular Biology, the Affiliated Cancer Hospital, School of Medicine, University of Electronic Science and Technology of China, Sichuan Cancer Hospital & Institute, No.55 Ren-min-nan Road, Chengdu, 610000 Sichuan Province People’s Republic of China; 4 Chengdu University of Traditional Chinese Medicine, Chengdu, 610032 Sichuan Province People’s Republic of China

**Keywords:** Pegylated liposomal doxorubicin, Cardiotoxicity, High-risk factors, Cardiac function test, Ovarian cancer

## Abstract

Pegylated liposomal doxorubicin (PLD) is a nano-doxorubicin anticancer agent. It was used as early as 2014 to treat ovarian and breast cancer, multiple myeloma and Kaposi's sarcoma. The 2018 National Comprehensive Cancer Network guidelines listed PLD as first-line chemotherapy for ovarian cancer. PLD has significant anticancer efficacy and good tolerance. Although PLD significantly reduces the cardiotoxicity of conventional doxorubicin, its cumulative-dose cardiotoxicity remains a clinical concern. This study summarizes the high-risk factors for PLD-induced cardiotoxicity, clinical dose thresholds, and cardiac function testing modalities. For patients with advanced, refractory, and recurrent malignant tumors, the use of PLD is still one of the most effective strategies in the absence of evidence of high risk such as cardiac dysfunction, and the lifetime treatment dose should be unlimited. Of course, they should also be comprehensively evaluated in combination with the high-risk factors of the patients themselves and indicators of cardiac function. This review can help guide better clinical use of PLD.

## Introduction

**Fig. 1:**
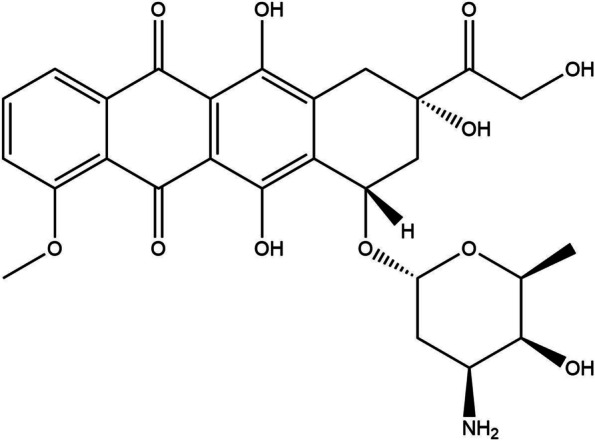
Chemical structure of doxorubicin

Doxorubicin (Dox) is an anthracycline compound with the molecular formula of C_27_H_29_NO_11_. Its chemical structure is shown in Fig. 1. It acts as a topoisomerase I inhibitor, can effectively inhibit the synthesis of DNA and RNA in tumor cells, and plays a cytotoxic role. In 1964, the Food and Drug Administration (FDA) clinically approved Dox for the treatment of a variety of cancers, such as ovarian cancer, thyroid cancer, stomach cancer, breast cancer, lymphoma, multiple myeloma, and sarcoma [1]. However, doxorubicin is reduced to semiquinone in the body; after the oxidation reaction, it can lead to the generation of free radicals and attack the myocardial mitochondrial membrane. Given that the myocardial ability to scavenge free radicals is low, myocardial toxicity occurs. The affinity of Dox to the myocardium is significantly higher than that to other tissues in the body, which makes the myocardium more vulnerable to Dox damage. Cardiotoxicity can manifest as acute or subacute damage immediately after treatment, or delayed cardiomyopathy after several years. Therefore, the mortality caused by Dox dose-dependent severe heart failure can be as high as 20%, which limits its clinical application [2].

Since 1971, when Gregoriadis and Rymen first reported the use of liposomes as drug carriers, liposomes have been used as effective carriers of anthracycline drugs. Liposomes are double-layer phospholipid membrane vesicles with similar biomembrane structures. Most of the liposomes cannot penetrate tissue capillaries, and the increased permeability of tumor tissue capillaries increases the local enrichment of drugs, thereby increasing their antitumor effect. Since 1995, liposome doxorubicin (Doxil), the first nano-particle–based antitumor drug approved by the FDA, has widely been used in the treatment of various tumors [3]. However, the clinical application of Doxil is also limited due to its poor structural stability, drug leakage, short storage life, poor tissue targeting, and easy clearance by the body.

**Fig. 2:**
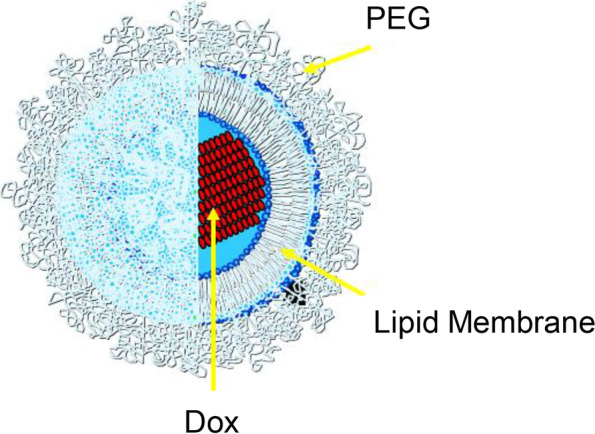
Chemical structure of pegylated liposomal doxorubicin

PEGylated liposomal doxorubicin (PLD) is a new type that wraps macromolecular substance Polyethylene glycol (PEG) on the surface of Doxil. PEG macromolecule can be covalently connected to the amino group of distearoyl phosphatidyl-ethanolamine (DSPE) (Fig. 2). It is composed of 45 ethylene glycol monomers and binds between 135 and 180 water molecules. This highly mobile and highly hydrated PEG macromolecule increases the stability of the Doxil spatial structure [4]. A previous study has shown that PLD accumulates in the liver, spleen, and tumor, but not in the heart tissue [5]. Dox in the heart tissue was only 38.1%. The release rate of Dox in the heart was much slower than that in other tissues, which could effectively reduce the cardiotoxicity of Dox. Therefore, PLD is widely used to treat metastatic breast cancer, ovarian cancer, progressive myeloma, refractory AIDS-related Kaposi's sarcoma (KS), and other solid tumors [6]. Although PLD can reduce the cardiotoxicity of Dox, its impact on the heart has not been fully revealed, and is still the focus of clinical attention.

### Strengths and limitations of pegylated liposomal doxorubicin in the treatment of ovarian cancer

The 2017 National comprehensive cancer network (NCCN) Guidelines recommended that carboplatin combined with PLD be added as one of the initial chemotherapy regimens for ovarian cancer. PLD plus carboplatin produced a better Progression-free survival (PFS) than standard-regimen Paclitaxel plus carboplatin and was well tolerated. Clinical study supported the continued use of PLD plus carboplatin as first-line chemotherapy for platinum-sensitive recurrent ovarian cancer, and recommend PLD at 30 mg/m^2^ every 4 weeks can be used as the initial dose. As a single-agent therapy, PLD manifested survival similar to other agents and was well tolerated. PLD monotherapy as first-line chemotherapy for platinum-resistant or -refractory recurrent ovarian cancer, and clinical recommend using PLD at a dose of 40 mg/m^2^ every 4 weeks as the initial dose [7–9].

The most concerning potential side effect of Dox and PLD is often cited as congestive heart failure (CHF), and doxorubicin is in fact closely associated with CHF. PLD’s parent drug is Dox, but PLD can effectively reduce cardiac toxicity.

### Pegylated liposomal doxorubicin reduces doxorubicin cardiotoxicity

The mechanism of cardiac injury is related to the production of free radicals. Free radicals induce peroxidation of muscle cell membrane, mitochondrial damage, and subsequent calcium inflow into cells, and the cytoplasm of isolated cells in myocardium becomes vacuolized. With the increase of Dox cumulative dose, more cells are involved, eventually leading to chronic and irreversible dilated cardiomyopathy and CHF.

A previous study found that the cardiotoxicity of Dox was related to the peak concentration of plasma Free-Dox [10]. The mechanism by which PLD reduces cardiac toxicity may be that PLD does not enter into tight capillary junctions like gastrointestinal tract and heart because of the size of liposomes[8], which results in less distribution of Dox in the myocardium [11]. Second, PEG macromolecule reduces the interaction between liposomes and blood component including opsonins and macrophages, maintains the liposome shape before Dox enters the tumor, and prolongs the circulation time of Dox. PEGylated coating of PLD forms a hydrophilic barrier protecting the liposomes from reticuloendothelial system detection and prolong the half-life of drugs in vivo [12]. Therefore, PLD leaves blood vessels much slower than Dox [13–, 14–16]. The prolongation of drug half-life significantly limits the peak value of Dox exposure to the myocardium, thereby reducing cardiac toxicity. Second, the release rate of PLD is also closely related to the gradient of ammonium sulfate in the tissue microenvironment [17]. In an additional study, it was demonstrated that PLD made with ammonium-methane sulfonate exhibit a much lower Hand and Foot syndrome (HFS) [18]. This will likely be the next research focus.

### Clinical cumulative dose threshold of pegylated liposomal doxorubicin

Dox resistance is multifactorial and involves a variety of cellular mechanisms, but at least some of these resistance mechanisms are reversible [19]. PLD has been proven to overcome P-glycoprotein (Pgp) mediated multidrug resistance [20]. Therefore, PLD may be an effective treatment option for recurrent cancer [21, 22]. For metastatic breast cancer, PLD can be used for re-treatment without cumulative toxicity [23]. PLD is the first-line chemotherapy drug for advanced KS. Due to the limited substitution therapy for KS, long-term multi-course PLD treatment is sometimes required. When the cumulative dose exceeds 500 mg/m^2^, there is little evidence to support stopping PLD treatment for patients with refractory HIV-related KS. Similar to conventional Dox, PLD cumulative dosing should not exceed 550 mg/m^2^ because of the risk of CHF [5]. Therefore, further studies are needed to determine the threshold of cumulative-dose cardiotoxicity of PLD in the treatment of advanced, refractory, and recurrent malignant tumors [24].

A randomized clinical trial reported that when the cumulative PLD dose reached 1061 mg/m^2^, less than 2% of patients developed nonfatal heart failure [25]. Other studies have found that patients with recurrent ovarian cancer who received a PLD cumulative dose of up to 2877 mg/m^2^ did not have a significant decline in cardiac function [26, 27]. Roswell Park Cancer Institute retrospectively studied ovarian cancer patients who had received a cumulative dose of PLD 6400 mg/m^2^ without interruption or discontinuation of treatment due to cardiotoxicity [28]. It has been confirmed that patients receiving PLD > 500 mg/m^2^ or PLD combined with previous Dox have no PLD-related heart failure after 10 years of follow-up [29].

When the dose exceeded 1110 mg/m^2^, the left ventricular ejection fraction (LVEF) decreased by more than 10% in 3.5% (5 cases) of patients; two of these cases were previously diagnosed as CHF, and a cumulative dose threshold of PLD 1000 mg/m^2^ was recommended for people at high risk [27]. Andreopoulo et al. studied PLD combined with other chemotherapy drugs. Among patients who had been treated for more than a year and whose cumulative dose reached 2460 mg/m^2^, only one patient developed transient heart failure when neutropenic sepsis related to topotecan administration occurred 10 months after stopping PLD [30]. Therefore, for people at high risk of cardiotoxicity, PLD cumulative dose should be reduced and cardiac function testing should be performed.

For patients with advanced, refractory, and recurrent malignant tumors, the use of PLD is still one of the most effective strategies in the absence of evidence of high risk such as cardiac dysfunction [23], and the lifetime treatment dose should be unlimited [25] (Table 1).

**Table 1:** Clinical trials with pegylated liposomal doxorubicin

Study	Intervention	Type of trial	Patient characteristics	Outcomes
Zhen Yuan 2021 [9]	PLD 40 mg/m^2^ q4wks 6 cycles	open-label, single-arm and multicenter prospective clinical trial	ovarian cancer	ORR, DCR, AEs, QOL
ALEX 2015 [26]	PLD 40 mg/m^2^ q4wks longer than 1 year	retrospective chart clinical trial	recurrent ovarian cancer, tubal and peritoneal carcinoma	PFS, OS, Cardiac Toxicity
Sarah E 2013 [27]	PLD 40 mg/m^2^ q4wks adjustments for toxicity consisted of either dose reduction or treatment delay	retrospective chart clinical trial	recurrent or refractory ovarian cancer, or endometrial cancer, primary peritoneal cancer, and fallopian tube cancer	Cardiac Toxicity
Joshua P 2010 [28]	PLD 30 or 40 mg/m^2^ q4wks adjustments for toxicity consisted of either dose reduction or treatment delay	retrospective chart clinical trial	ovarian cancer, primary peritoneal, endometrial, fallopian, tube, cervix and vulva cancer	Cardiac Toxicity
E. Andreopoulou 2008 [30]	PLD 30 or 40 mg/m^2^ q4-8wks adjustments for toxicity consisted of either dose reduction or treatment delay	retrospective chart clinical trial	recurrent ovarian cancer, fallopian tube cancer	Cardiac Toxicity
M. E. R. O’Brien 2004 [31]	PLD 50 mg/m^2^ q4wks	phase III randomized multicenter, open-label trial	women with metastatic breast cancer	PFS, OS, Cardiac Toxicity
	doxorubicin 60 mg/m^2^ q3wks			
Denise Uyar 2004 [32]	PLD 20 or 40 mg/m^2^ q4-6wks ≥ 6 cycles	retrospective chart clinical trial	ovarian cancer, primary peritoneal and endometrial cancer	Cardiac Toxicity
Sandrine Faivre 2004 [33]	PLD 35 mg/m^2^ q3wks	phase I–II randomized multicenter, open-label trial	recurrent squamous cell carcinoma of the head and neck	ORR, Toxicity
	PLD 45 mg/m^2^ q3wks			
C.L. Kushnir 2015 [34]	PLD cumulative doses of 300 mg/m^2^ (range 60–1420 mg/m^2^)	retrospective chart clinical trial	ovarian cancer, primary peritoneal, Fallopian tube, endometrium, cervix, GYN origin, ovary and endometrium, Vaginal cancer	Cardiac Toxicity
Keith M 2017 [35]	cumulative doxorubicin > 450 mg/m^2^ (free doxorubicin combined with PLD),25% patients > 1000 mg/m^2^	retrospective chart clinical trial	advanced malignancies	Cardiac Toxicity
G. Berry 1998 [36]	cumulative PLD (20 mg/m^2^ q2wks) of 440—840 mg/m^2^	retrospective chart clinical trial	AIDS Kaposi's sarcoma	Cardiac Toxicity
	cumulative doxorubicin (20 mg/m^2^ q2wks) of 174–671 mg/m^2^			
Cardiac Toxicity 2004 [37]	cumulative doxorubicin dose of ≥ 550 mg/m^2^ ( including PLD) or ≥ 400 mg/m^2^ of PLD alone	retrospective chart clinical trial	advanced malignancies	Cardiac Toxicity
Sarah E.2013 [38]	PLD median dose of 200 mg/m^2^ (range 40 -1775 mg/m^2^)	retrospective chart clinical trial	ovarian cancer, endometrial and other cancer	Cardiac Toxicity

*ORR* Objective response rate, *DCR* Disease control rate, *AEs* Adverse events, *QOL* Quality of life, *PFS* Progression-free survival, *OS* Overall survival, *AIDS* Acquired immune deficiency syndrome

### High risk population for pegylated liposomal doxorubicin induced cardiotoxicity

High interindividual variability based on age and sex and cardiotoxicity related to plasma pharmacokinetics are unpredictable [39]. For high-risk groups (previous chest wall/mediastinal radiotherapy history, elderly women, previous diagnosis of congestive heart failure, subjective symptoms, or clinical evidence of cardiotoxicity), cardiac function testing and reduction of cumulative dose are necessary.

### Elderly individuals

For elderly patients, the use of PLD adjuvant chemotherapy has attracted attention [31]. It has been shown that during PLD treatment, three patients had cardiac symptoms that may be related to PLD or aggravated by PLD [32]. These patients were more than 65 years old. Because elderly patients often have chronic complications, there are a variety of cardiac risk factors, such as uncontrolled high blood pressure, history of myocardial infarction, aortic stenosis, and arrhythmia, so older age is one of the high-risk factors of PLD dose [40].

### Women

Male patients with solid tumors or KS show twofold accelerated plasma clearance compared with women of all ages [41]. In a clinical study of female patients with advanced breast cancer, patients who received PLD at a cumulative dose of 450–550 mg/m^2^ had an 11% risk of cardiotoxicity. For ovarian cancer, the high cumulative dose of PLD exceeds the total lifetime cumulative dose of 550 mg/m^2^ initially recommended by FDA, but there is a lack of evidence to support the standard practice guidelines for these patients [12].

Compared with the general population, the cardiovascular mortality of female patients with breast cancer is increased, and the risk is about twice as high when considering age, menopausal status, and other typical risks [15]. The increased cardiovascular incidence rate in female patients with breast cancer is due to the frequent occurrence of adverse classic risk factors that are usually not optimally controlled (such as smoking, low physical activity, high body mass index, dyslipidemia) and the adverse effects of cancer treatment [42]. If women have other risk factors, such as diabetes or hypertension, Dox therapy brings a higher risk of cardiovascular death after breast cancer [43, 44]. Although this source of variability is not considered in the current PLD administration guidelines, it is necessary to be cautious in the clinical application [6].

### Child

Children may be more susceptible to the cardiac effects of anthracyclines compared with adults [45]. Genetic variations seem to be associated with the development of Dox-induced cardiotoxicity in children, so the current PLD research findings may not be applicable to the treatment of children with cancer [46].

### Previous heart disease and chest wall/mediastinal radiotherapy history

In a study on PLD treatment for women with recurrent ovarian or peritoneal cancer, the cumulative dose of PLD was more than 550 mg/m^2^, and the median follow-up time was 20 months. Among them, 53 patients had obvious preexisting cardiac risk factors (previous cardiomyopathy, previous chest wall/mediastinal radiotherapy) and received initial cardiac assessment or monitoring. Only three patients (1.6%) developed CHF that may be related to PLD treatment.

Doxil might protractedly release doxorubicin in interstitial areas as liposomes diffuse slowly into the tumor tissue [47]. Prior high doses of radiotherapy might facilitate this process since late radiation induced tissue modifications, including fibrosis, would establish intratumoural conditions that rapidly breakdown the extravasated liposomes, facilitating doxorubicin radiation recall effects, slowing down healing processes, and inducing tumor necrosis [48]. Therefore, this drug needs to be used carefully for tumors relapsing in irradiated areas [33]. In radiotherapy for breast cancer, left breast radiotherapy is also associated with a higher risk of cardiovascular death and myocardial infarction death than right breast radiotherapy [49]. For patients undergoing concurrent radiotherapy and chemotherapy, the clinical application of PLD requires paying attention to these related risks.

### Doxorubicin in childhood

Dox induced cardiotoxicity can be delayed, especially in cancer survivors treated during childhood. Among these cancer survivors, the incidence rate of heart failure and other heart diseases is much higher than the incidence rate in the general population [34]. In these patients, cardiac function should be monitored if PLD treatment is needed again in adulthood.

### Cardiac function monitoring method

#### Left ventricular ejection fraction

FDA recommends routine assessment of LVEF by multi-gated radionuclide angiography (MUGA) or echocardiography before, during, and after PLD treatment [12]. The decrease in LVEF is regarded as a biomarker that is related to heart failure or can predict the development of heart failure [50]. Of note, 11% is the minimum change in LVEF, and the recognizable confidence is 95% [51]. The most sensitive cardiac function monitoring method is continuous measurement of LVEF. Gill et al. suggested that baseline LVEF may be sufficient to determine the overall cardiac risk during PLD treatment [23]. If the baseline LVEF is less than 30%, the use of such drugs is not recommended. When the baseline LVEF is above 30% but below 50%, patients can still receive treatment, but the LVEF needs to be measured repeatedly before each course of treatment. For patients with baseline LVEF above 50%, routine cardiac testing can be ignored [34].

However, some scholars believe that although LVEF is widely used to monitor the cardiotoxicity of Dox, and there are various guidelines and recommendations, the use of LVEF is not without shortcomings [35]. Clinically, there are also heart failure patients with normal ejection fraction (LVEF > 50%). Because the significant decrease in LVEF does not necessarily occur gradually, but may occur suddenly. In normal subjects, LVEF may change moderately every day depending on hydration status. Transient changes in LVEF that are not related to Dox are not uncommon in clinical practice, and may lead to premature cessation of treatment before reaching the maximum recommended cumulative dose. Therefore, the utility of LVEF changes in predicting Dox-induced heart failure has not been fully demonstrated, and it may not be the best objective indicator [35].

#### Doppler-based myocardial deformation imaging

Early cardiac function changes induced by chemotherapy are subtle, and LVEF is too insensitive to detect subtle changes in cardiac function [52]. Velocity, strain, and strain rate measurements based on Doppler myocardial imaging have been shown to be sensitive in quantifying cardiac dysfunction in other situations. Compared with conventional echocardiography and myocardial velocity measurement, myocardial deformation parameters (S and SR) allow for the detection of subtle changes in left ventricular longitudinal and radial function after six cycles of PLD. We recommend Doppler-based myocardial deformation imaging (DMI) for monitoring cardiac function during chemotherapy [53–56].

#### Endomyocardial biopsy

Berry et al. consider endomyocardial biopsy (EMB) as the gold standard [36]. Biopsy grade is predictive of the rate of progression of cardiotoxicity and is considered the most sensitive indicator of conventional doxorubicin-induced cardiotoxicity. A study confirmed that the Billingham score of all patients with HIV-related KS, breast cancer, and ovarian cancer with PLD > 500 mg/m^2^ (500–1485 mg/m^2^) was lower than 1.5, indicating that none of the patients had significant histological myocardial damage [24]. In another study, two (25%) patients with advanced malignancies underwent two EMB. Both biopsy scores were 1.5. This suggests that additional exposure to 420 mg/m^2^ of PLD between the first and second biopsies was not associated with additional cardiac damage. But these patients both received previous conventional Dox therapy (360 mg/m^2^) and chest irradiation, both of whichare associated with an increased risk of anthracycline-induced cardiac damage [37].

## Conclusions

Most patients receiving PLD treatment suffer from advanced, refractory, or recurrent malignant tumors. Quality of life and symptom relief are their main concerns [38]. The commonly used cardiac function testing methods in clinical practice have advantages and disadvantages. It is suggested that they can only be used for auxiliary evaluation, and they cannot directly predict the cardiac toxicity of PLD treatment. Overall, PLD cardiac safety is good and there is no absolute upper limit for clinical cumulative dose. They also should be comprehensively evaluated in combination with the high-risk factors of the patients themselves and the variability between individuals.

## Data Availability

Not applicable.

## Abbreviations

AIDS: Acquired Immune Deficiency Syndrome
AEs: Adverse events
CHF: Congestive heart failure
Dox: Doxorubicin
DCR: Disease control rate
DSPE: Distearoyl-phosphatidylethanolamine
DMI: Doppler based myocardial deformation imaging
EMB: Endomyocardial biopsy
FDA: Food and Drug Administration
HFS: Hand and Foot syndrome
KS: Kaposi's sarcoma
LVEF: Left ventricular ejection fraction
Doxil: Liposome doxorubicin
MUGA: Multi gated radionuclide angiography
NCCN: National comprehensive cancer network
ORR: Objective response rate
OS: Overall survival
PLD: Pegylated liposomal doxorubicin
PFS: Progression free survival
Pgp: P-glycoprotein
PEG: Polyethylene glycol
QOL: Quality of life
